# Toward Sustainable Pesticide Application: Bridging Efficacy and Food Safety

**DOI:** 10.1002/advs.202516248

**Published:** 2025-10-30

**Authors:** Xuan Li, Janar Tursen, Wanbin Zhu, Xufeng Yuan, Hongliang Wang

**Affiliations:** ^1^ Center of Biomass Engineering/College of Agronomy and Biotechnology China Agricultural University Beijing 100193 China; ^2^ Sanya Institute of China Agricultural University Sanya 572025 China

**Keywords:** coacervate, environmental risk, food safety, lignin, pesticide delivery

## Abstract

Improving the adhesion and spreading of pesticides on plant surfaces is essential for maximizing efficacy, yet current approaches often exacerbate pesticide residues, posing risks to environmental and food safety. Sustainable alternatives are urgently needed to enhance pesticide utilization while minimizing collateral harm. Here, a fully bio‐based pesticide delivery system is presented constructed from lignin coacervates—formed via hydrophobic and electrostatic interactions between aminated alkaline lignin and sodium lignosulfonate, both readily available industrial byproducts—and used for efficient encapsulation the hydrophobic pesticide abamectin. The resulting formulation improves pesticide retention on leaves, enhances resistance to ultraviolet degradation, and ensures strong adhesion and wetting, critical factors for reducing spray drift and runoff. Importantly, the delivery system enables pH‐ and laccase‐triggered release, aligning with the biological environment of target pests and supporting controlled, prolonged action. By physically shielding the active ingredient, the coacervates reduce non‐target exposure and ecological toxicity by a factor of two to three. Moreover, the formulation demonstrates superior removability under household washing, as it can be completely eliminated with only a few simple rinses, thereby substantially lowering the risk of foodborne residues. Overall, this work establishes a sustainable pesticide‐delivery platforms that reconciles agricultural efficacy with environmental and food safety imperatives.

## Graphical abstract

A fully bio‐based lignin coacervate system is developed for pesticide delivery, enhancing leaf retention, UV resistance, adhesion, and wetting. The formulation enables pH/laccase‐triggered release for prolonged action, reduces ecological toxicity, and is fully washable, reconciling agricultural efficacy with food and environmental safety.

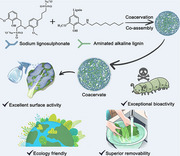

## 1 Introduction

Feeding a growing global population demands increased agricultural productivity, both in yield and quality. Pesticides remain indispensable for effective pest management.^[^ ^1^, ^2^ ^]^ However, conventional application methods suffer from extremely low utilization efficiency, less than 1% of active ingredients reach target pests.^[^ ^3^ ^]^ Most are lost through volatilization, photodegradation, leaching, drift, and especially poor adhesion on hydrophobic plant surfaces.^[^ ^4^, ^5^ ^]^ Splashing and rebound of droplets from leaf cuticles are major contributors to this inefficiency, leading to economic losses, environmental contamination, and human exposure risks.^[^ ^6^, ^7^ ^]^ In response, formulation technologies have advanced to improve spray retention and leaf deposition, often through enhanced spreading and adhesion.^[^ ^8^, ^9^ ^]^ Yet such strategies can result in tightly bound residues that persist on food surfaces and resist removal by typical household washing, raising serious food safety concerns.^[^ ^10^ ^]^ Therefore, next‐generation pesticide delivery systems must strike a balance between high efficacy in the field and effective removability after harvest, while aligning with broader sustainability goals.

Micro‐ and nanoscale delivery systems, including nano‐emulsions, micelles, particles, and metal–organic frameworks, have shown promise in enhancing pesticide performance.^[^ ^11^, ^12^, ^13^ ^]^ However, their agricultural scalability remains constrained by complex synthesis routes, high material costs, uncertain long‐term toxicity, and potential environmental burdens.^[^ ^14^ ^]^ Coacervate‐based delivery platforms offer a compelling alternative.^[^ ^15^, ^16^ ^]^ Coacervates are dense, liquid‐like droplets that form in aqueous solutions through the electrostatic complexation of oppositely charged polymers or macromolecules. This process, known as liquid‐liquid phase separation, creates a polymer‐rich coacervate phase that coexists with a dilute aqueous phase. ^[^ ^17^ ^]^ They offer a versatile, solvent‐free medium for encapsulating active compounds with tunable release profiles, while simultaneously enhancing spray stability and surface interactions.^[^ ^18^ ^]^ Despite these advantages, most existing coacervate systems rely on proteins, nucleic acids, or synthetic surfactants that are expensive, difficult to scale, or environmentally problematic. Developing coacervates from inexpensive, naturally derived components would provide a more sustainable pathway, but presents significant design challenges.

Lignin, the second most abundant biopolymer on Earth, is an industrial byproduct generated in over 60 million tons annually through pulping and biomass refining processes.^[^ ^19^, ^20^ ^]^ Despite its availability and low cost, lignin remains vastly underutilized.^[^ ^21^, ^22^ ^]^ Its aromatic structure features diverse functional groups, such as phenolic hydroxyls, carbonyls, and methoxy groups, that offer rich chemical tunability.^[^ ^23^, ^24^ ^]^ Through modifications like sulfonation and amination, lignin derivatives can acquire amphiphilic properties conducive to self‐assembly.^[^ ^25^ ^]^ Moreover, lignin is a natural UV absorber, owing to its chromophoric phenolic and ketone groups, making it particularly suitable for protecting UV‐sensitive active ingredients.^[^ ^26^, ^27^ ^]^ Its amphiphilic character, with hydrophobic aromatic domains and hydrophilic functional groups, also grants it excellent interfacial activity.^[^ ^28^, ^29^ ^]^ Collectively, these attributes position lignin as a promising and environmentally compatible building block for constructing bio‐based coacervate systems.

In our previous work, we demonstrated that aqueous coacervates formed from sodium lignosulfonate (SL) and surfactants, such as surfactin or synthetic amphiphiles, can encapsulate both hydrophilic and hydrophobic molecules, while providing strong UV protection and excellent foliar wettability.^[^ ^30^ ^]^ While lignin‐based particles and microcapsules have been explored for agrochemical delivery, they often rely on energy‐intensive manufacturing, synthetic polymers, or organic solvents. Similarly, previously reported coacervate systems for agricultural use frequently depend on proteins, chitosan, or synthetic polyelectrolytes. Meanwhile, constructing coacervates solely from lignin derivatives, without reliance on proteins or synthetic agents, remains an unsolved challenge. More importantly, such systems must reconcile high field performance with reduced residue persistence to meet environmental and consumer safety expectations.

In this study, the system we present here is distinguished by its fully bio‐based, solvent‐free, and entirely lignin‐derived nature to address these challenges. Sodium lignosulfonate (SL) serves as the anionic component, while aminated alkaline lignin (AAL), synthesized via functionalization with amines of varying chain lengths, provides the cationic counterpart. These components form coacervates in water without the use of organic solvents or synthetic additives. We demonstrate the formulation's capability to encapsulate the hydrophobic pesticide abamectin (AVM), while investigating the influence of amine chain length on coacervate formation, stability, and performance. The coacervates were characterized by FTIR, SEM, optical microscopy, and confocal imaging. Binding interactions were probed through isothermal titration calorimetry, zeta potential analysis, and conductivity measurements. We further evaluated the encapsulation efficiency, UV resistance, leaf adhesion, and pH/laccase‐responsive release of AVM, alongside its insecticidal activity. Importantly, we assessed residue removability under household washing and the environmental safety of the formulation in non‐target organisms, including zebrafish, earthworms, and soil microbes. This work presents a scalable and bio‐based strategy for designing multifunctional pesticide delivery systems that achieve high agrochemical efficacy while minimizing ecological and food safety risks.

## 2 Results and Discussion

### 2.1 Preparation of AAL‐SL Coacervates

The construction of complex coacervate droplets via electrostatic and hydrophobic interactions between biopolymer‐based components presents a sustainable and versatile platform for environmentally responsive delivery systems.^[^ ^31^ ^]^ In this study, we systematically examined the physicochemical interactions underpinning coacervate formation between anionic lignosulfonate (SL) and cationic aminated alkaline lignin (AAL), with the latter functionalized by alkylamines of varying chain lengths. SL, a water‐soluble lignin derivative obtained from sulfite pulping, possesses multiple sulfonic acid groups (─SO^3−^) covalently bound to an aromatic backbone, resulting in a high negative charge density under near‐neutral pH conditions.

To investigate how hydrophobicity and alkyl chain length influence coacervation behavior, we synthesized a series of cationic lignin derivatives by grafting primary monoalkylamines (C_1_–C_8_) onto alkaline lignin via a Mannich‐type reaction. This reaction proceeds through the condensation of formaldehyde, alkylamines, and phenolic hydroxyl groups on lignin under mild alkaline conditions, yielding aminomethylated side chains anchored at activated aromatic positions. The resulting AAL variants, designated AAL_1_, AAL_2_, AAL_3_, AAL_4_, and AAL_8_, exhibited systematically increasing hydrophobicity while maintaining comparable levels of cationic charge.

**Scheme 1:**
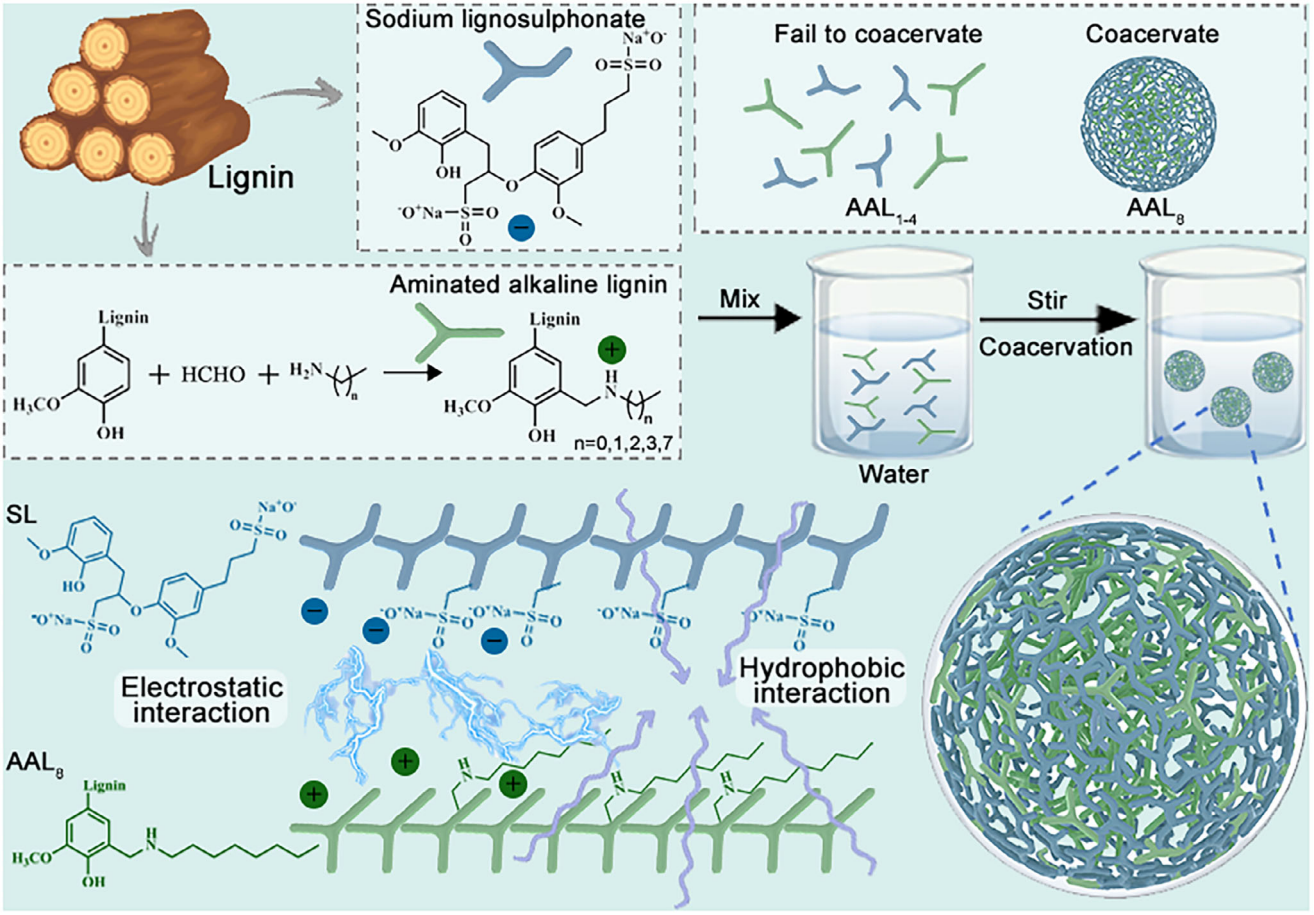
Scheme of amination modification of alkaline lignin and formation of coacervate droplets.

Among the synthesized derivatives, only AAL_8_, containing the longest alkyl chain (C_8_), was capable of forming stable coacervate droplets with SL (Figure S5, Supporting Information), probably through electrostatic and hydrophobic interactions, as shown in **Scheme** **1**. In contrast, AALs modified with shorter alkyl chains (C_1_–C_4_) failed to produce stable coacervates under the same conditions (Figures S1–S4, Supporting Information). This observation highlights that electrostatic interaction alone is insufficient to drive phase separation. A critical threshold of hydrophobic interaction, conferred by the alkyl chain, is also necessary to enable complex coacervation.

Successful grafting in AAL_8_ was confirmed by multiple spectroscopic and analytical techniques. A new FTIR absorption peak at 1631 cm^−1^ corresponds to alkylamino groups (Figure S6a, Supporting Information). Elemental analysis revealed increased nitrogen and hydrogen content (Table S1, Supporting Information), and a characteristic aminomethyl proton signal appeared at 3.31 ppm in the ^1^H NMR spectrum (Figure S6b, Supporting Information). Upon mixing AAL with SL in aqueous solution, the oppositely charged polymer chains undergo spontaneous complexation through coulombic attraction between sulfonate groups on SL and protonated amine groups on AAL. This leads to the formation of dynamic interpolymer complexes in solution. The subsequent phase behavior is strongly dependent on the length of the grafted alkyl chain. In the case of AAL_8_, the C_8_ chains introduce sufficient hydrophobicity to form water‐excluding microdomains that promote interchain aggregation. These interactions, together with van der Waals forces between the alkyl chains and *π*–*π* stacking among lignin aromatic rings, stabilize the condensed coacervate phase.

**Figure 1:**
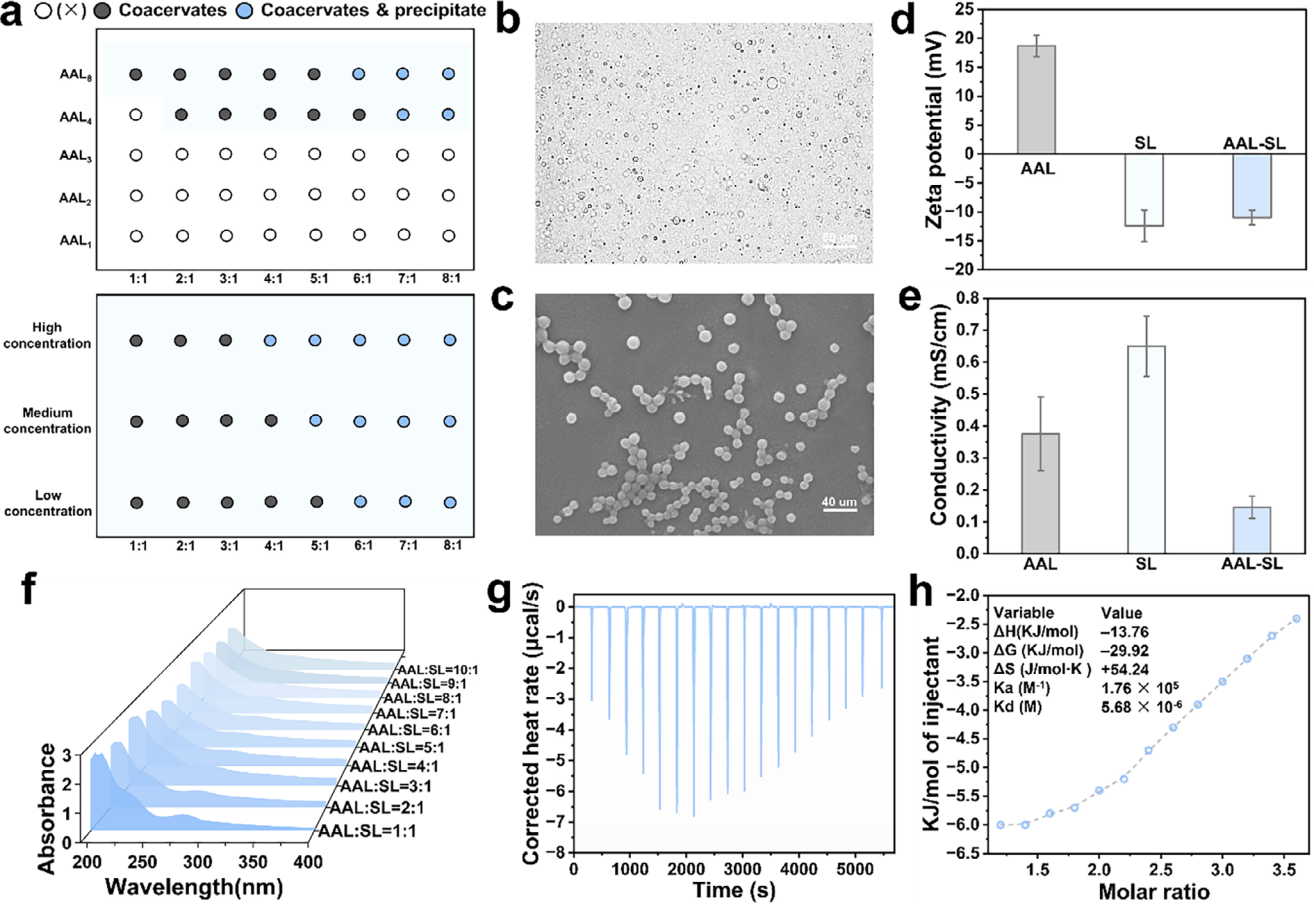
Phase diagrams a) and Bright field microscopy b) of AAL‐SL coacervates; SEM images of AAL‐SL coacervates after natural drying c); Zeta potential d) and conductivity e) of AAL‐SL coacervates; UV absorption spectra of different mass ratios of combined AAL and SL f); Isothermal titration calorimetry curves for the action of AAL with SL g,h).

In the **Figure** **1**a, the phase transition behavior was further illustrated by a phase diagram constructed by varying the mass ratios of AAL_8_ to SL. As the mass ratio of AAL_8_:SL increased, the system transitioned from a homogeneous single‐phase solution to a biphasic coacervate system. Turbidity measurements supported this transition, with a sharp increase in light scattering observed at the phase boundary (Figure S7, Supporting Information). At higher concentrations, the system underwent further demixing, leading to macroscopic precipitation likely caused by excessive interpolymer association and loss of colloidal stability. In contrast, shorter‐chain AALs (C_1_ to C_4_), although positively charged, lack sufficient hydrophobic character to induce phase separation. The resulting electrostatic complexes remain dispersed as soluble macromolecular assemblies, likely forming small, non‐coalescent aggregates or remaining molecularly dissolved. The key distinction lies in the amphiphilic structure of AAL_8_, which combines a hydrophilic head group (protonated amine) with a hydrophobic tail (C_8_ chain), mimicking classical surfactants and enabling self‐assembly in aqueous environments.

Notably, AAL_8_‐SL coacervates can be formed in the absence of inorganic salts. This is significant, as many synthetic polyelectrolyte systems require salt to screen repulsive interactions or to fine‐tune phase behavior. Here, the inherent hydrophobic and aromatic interactions in lignin eliminate the need for external ionic strength, thereby enhancing the biocompatibility and environmental sustainability of the system. In summary, lignin‐based coacervate formation relies on a cooperative mechanism involving electrostatic complexation and hydrophobic self‐assembly. While Coulombic attraction initiates the interaction between SL and AAL, only alkyl chains of sufficient length, such as octyl groups, enable phase separation into well‐defined droplets. These findings emphasize the critical role of molecular amphiphilicity in the design of bio‐based coacervate. This dual‐interaction model, combining charge‐driven complexation with chain‐length‐dependent hydrophobic aggregation, presents a versatile approach for engineering lignin coacervates with tunable properties.

#### 2.1.1 UV Absorption Spectra

The UV absorption spectra of coacervate droplets formed at varying AAL‐to‐SL mass ratios are shown in Figure 1f. As the proportion of AAL increased, the intensity of the characteristic UV absorption peak of the AAL–SL coacervates initially decreased, reaching a minimum at a mass ratio of 5:1. Beyond this ratio, further increases in AAL content led to a resurgence in absorption intensity. This trend suggests that at a 5:1 mass ratio, AAL and SL interact in an optimal stoichiometric ratio to form highly compact coacervates, effectively shielding the chromophoric groups of lignin from UV irradiation.^[^ ^32^ ^]^ At higher AAL levels, excess unbound AAL may contribute additional UV‐absorbing species, resulting in increased absorbance.

#### 2.1.2 Isothermal Titration Calorimetry

To elucidate the coacervation mechanism, isothermal titration calorimetry (ITC) was employed to characterize the thermodynamics of the interaction between AAL and SL (Figure 1g,h). The binding process was found to be predominantly driven by hydrophobic interactions. The observed exothermic enthalpy change (ΔH = −13.76 kJ mol^−1^) suggests that the hydrophobic alkyl chains of octylamine‐modified AAL engage in van der Waals interactions with the hydrophobic domains of SL, releasing thermal energy. Concurrently, electrostatic interactions between the sulfonic acid groups of SL and the secondary amines of AAL orient these moieties at the interface, further stabilizing the assembly and lowering the overall system energy. A substantial increase in entropy (ΔS = +54.24 J mol^−1^·K) reflects the release of structured water molecules from the hydration shells surrounding the hydrophobic regions upon coacervate formation, a hallmark of hydrophobic association. The resulting negative Gibbs free energy change (ΔG = −29.92 kJ mol^−1^) confirms that the process is thermodynamically favorable and spontaneous. Moreover, the high binding constant (K_a_ = 1.76 × 10^5^ M^−1^) underscores the strong affinity between AAL and SL, supporting the formation of a densely packed coacervate phase.^[^ ^33^ ^]^

#### 2.1.3 Optical Photothermal IR Microspectroscopy

**Figure 2:**
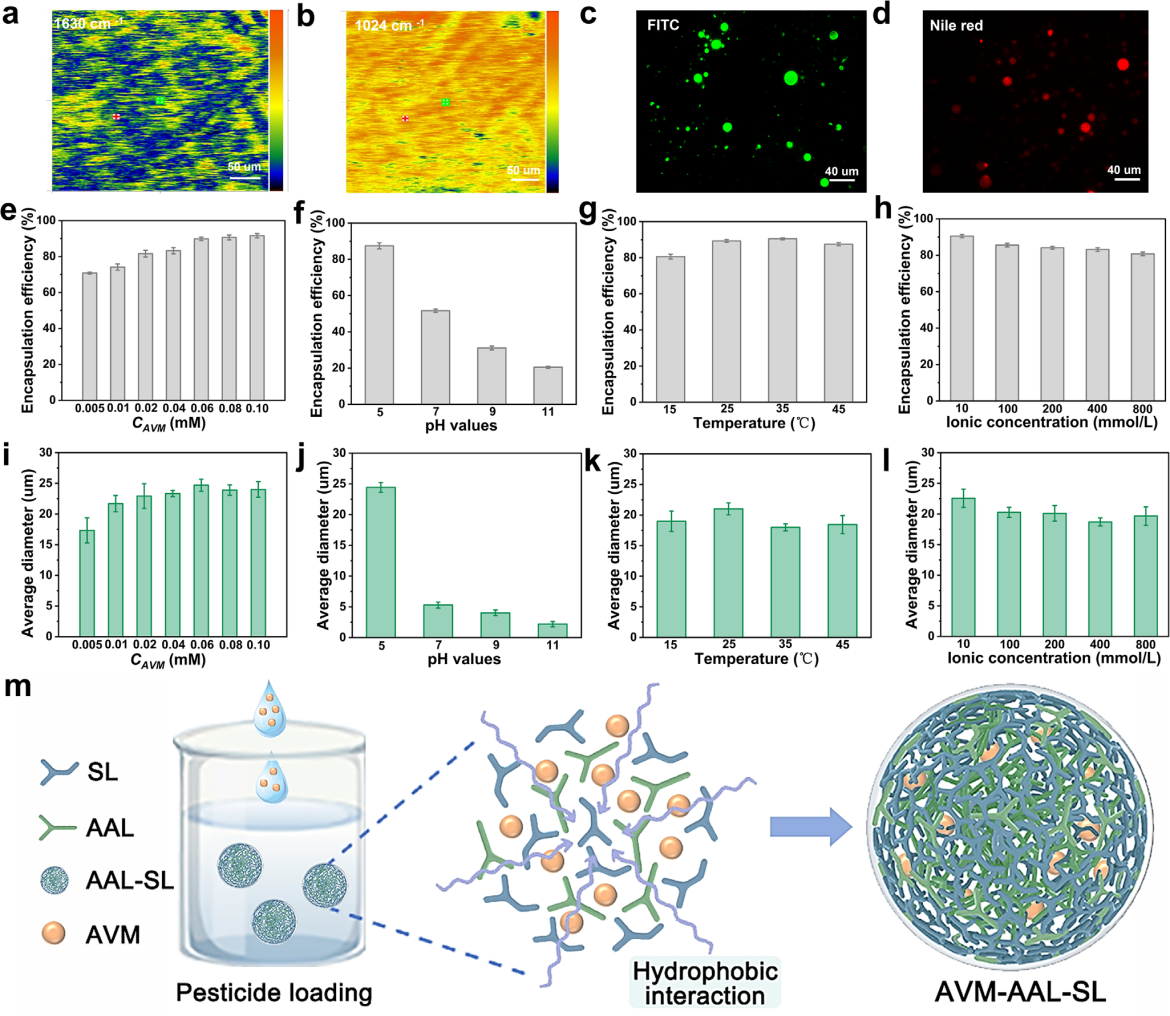
O‐PTIR photomicrograph of AAL‐SL coacervates a,b); Confocal fluorescence images of AAL‐SL coacervates with FITC or Nile red c,d); Effects of *C_AVM_* e), pH f), temperature g), and ionic concentration h) on encapsulation efficiency; Effects of C*_AVM_* i), pH j), temperature k), and ionic concentration l) on average diameter. The encapsulation mechanism of AVM m).

To gain spatially resolved molecular insights into the nanoscale architecture of coacervate droplets formed by the interaction of AAL_8_ and SL, we employed optical photothermal IR microspectroscopy. This label‐free technique allows in situ chemical mapping with high spatial and spectral fidelity, revealing how molecular constituents organize within soft matter assemblies. In the **Figure** **2**a,b, IR imaging identified well‐defined spherical microdomains with diameters of ≈12 µm, exhibiting strong and spatially co‐localized absorption at 1630 and 1024 cm^−1^. The 1024 cm^−1^ peak is characteristic of the sulfonate (─SO^3−^) symmetric stretching vibration, indicative of the SL component. The 1630 cm^−1^ band corresponds to N─H bending vibrations introduced via amine functionalization of AAL_8_. The pronounced co‐localization of these two absorption bands within the same spherical droplets confirms the formation of hybrid coacervates composed of both cationic AAL_8_ and anionic SL. The homogeneous spatial distribution of these signals suggests a well‐mixed internal phase, rather than a phase‐separated or core‐shell structure, indicating intimate molecular‐level interactions.^[^ ^34^ ^]^ These findings provide direct spectroscopic evidence that the formation of lignin‐based coacervate droplets is governed by electrostatic attraction between oppositely charged species. The deployment of confocal IR imaging thus offers a powerful means of visualizing and deconvoluting the complex interplay of forces that drive self‐assembly in bio‐derived coacervate.

### 2.2 Morphology of AAL‐SL Coacervates

As observed in Figure 1b, the resulting droplets display a spherical morphology under optical microscopy, undergo liquid‐like fusion, and exhibit internal dynamic rearrangement characteristic of coacervate systems. Scanning electron microscopy (SEM) image of AAL‐SL coacervate droplets obtained under ambient drying conditions reveal that the dried structures maintain a regular, spherical morphology (Figure 1c). This preservation of shape suggests that the original droplets possessed a liquid‐like internal structure and that phase separation occurred in a uniform and stable manner.

To investigate the partitioning behavior of solutes within the coacervate phase, we examined the ability of the droplets to sequester both hydrophilic and hydrophobic fluorescent probes. In the Figure 2c,d, the coacervates demonstrated substantial enrichment of fluorescein isothiocyanate (FITC), a representative hydrophilic dye, as well as Nile red, a hydrophobic fluorescent probe. This dual enrichment capability indicates the presence of both hydrophilic and hydrophobic microenvironments within the condensed phase. Such compartmentalization is likely driven by the amphiphilic character of the AAL‐SL, where sulfonate and amine functional groups contribute to hydrophilic domains, while alkyl chains and aromatic moieties derived from lignin promote hydrophobic interactions. The ability of these coacervate droplets to concurrently encapsulate solutes with diverse polarity profiles suggests broad applicability for pesticide delivery. Specifically, the system is capable of accommodating both water‐soluble agrochemicals and hydrophobic actives, such as abamectin, thereby enabling the development of multifunctional delivery platforms for modern agricultural applications.

### 2.3 Physicochemical Characteristics of AAL‐SL Coacervates

#### 2.3.1 Zeta Potential and Conductivity

As shown in Figure 1d, zeta potential measurements confirmed the presence of oppositely charged components in the coacervate system. Octylamine‐modified AAL exhibited a strongly positive surface potential (+18.67 mV), while SL, rich in sulfonic acid groups, displayed a negative potential (−12.40 mV). Upon complexation, the AAL–SL coacervates showed a net zeta potential of −10.97 mV, suggesting that hydrophilic sulfonate groups remained partially exposed at the interface. This residual surface charge likely facilitates colloidal stability by promoting electrostatic repulsion among coacervate droplets in aqueous media. Conductivity measurements (Figure 1e) further supported the interaction between the two components. SL exhibited a relatively high conductivity (0.65 mS cm^−1^) due to the dissociation of sulfonate groups, while AAL showed a moderate value (0.38 mS cm^−1^), attributable to the partial ionization of phenolic hydroxyls. After coacervate formation, the conductivity of the AAL–SL system dropped markedly to 0.15 mS cm^−1^, indicating electrostatic neutralization and a reduction in free ionic species. In addition, the formation of large, organized molecular aggregates may further impede ion mobility, contributing to the observed decrease.

### 2.4 Encapsulation of Hydrophobic Pesticide

The encapsulation efficiency of AAL‐SL for the hydrophobic pesticide AVM increased with the concentration of AVM (Figure 2e). At an AVM concentration of 0.005 mm, the encapsulation efficiency was 70.80%, and it increased to 91.57% when the concentration was raised to 0.10 mm. The partitioning of hydrophobic AVM molecules into the hydrophobic phase of the coacervate is a thermodynamically spontaneous process driven by the concentration gradient. A higher initial AVM concentration provides a stronger driving force, enabling more molecules to incorporate efficiently into the co‐condensate phase. In addition, once AVM is encapsulated, it further enhances the internal hydrophobicity of the coacervate microenvironment, thereby lowering the interfacial free energy and reinforcing the thermodynamic favorability for subsequent AVM partitioning. This self‐amplifying effect allows the coacervate to dynamically expand its hydrophobic domains until reaching the physical saturation limit, which explains why higher AVM concentrations result in higher encapsulation efficiency. These results demonstrate that the AAL‐SL system possesses excellent pesticide‐loading scalability, making it suitable for pesticide applications in various scenarios, thus providing a flexible regulatory window for formulation engineering. The encapsulation efficiency of AVM‐AAL‐SL progressively increased with rising AVM concentration, reaching a plateau at 0.06 mM, beyond which the average diameter exhibited no significant change, possibly due to the limited capacity of the hydrophobic regions within the coacervate droplet (Figure 2i). As shown in Figure 2f,j, both encapsulation efficiency and average droplet diameter reached their maximum at pH 5. Under neutral and alkaline conditions, these values declined significantly, with the decline becoming more pronounced at higher pH. This trend is likely due to the fact that acidic environments promote electrostatic and hydrophobic interactions, whereas alkaline conditions disrupt charge balance and degrade the lignin structure, thereby impairing coacervate formation. Additionally, temperature variations between 15 °C and 45 °C resulted in no significant changes in either encapsulation efficiency or average droplet diameter, indicating that the coacervates possess broad thermal adaptability (Figure 2g,k). Similarly, increasing the external ionic strength had minimal impact on both parameters, suggesting that ion shielding plays a limited role in these predominantly hydrophobically driven systems (Figure 2h,l).^[^ ^30^ ^]^ The small error bars indicated the accuracy of the experimental results. Collectively, these findings indicate that AVM‐AAL‐SL exhibits strong environmental resilience, supporting its potential for practical applications under fluctuating field temperatures, rainfall exposure, and varying water quality conditions.

As shown in Figure 2m, the encapsulation of AVM within AAL‐SL is a spontaneous process driven primarily by hydrophobic interactions, supported by complementary intermolecular forces. AVM's strong affinity for the hydrophobic microenvironments in octylamine‐modified AAL facilitates its incorporation via van der Waals interactions with alkyl chains and aromatic lignin domains. Electrostatic attraction between protonated amine groups of AAL and sulfonate groups of SL stabilizes the coacervate structure, while potential π–π stacking enhances binding. This entropy‐driven process, initiated by the collapse of hydration shells, enables efficient AVM partitioning into the coacervate phase, resulting in high loading and encapsulation efficiency.

### 2.5 Pesticide–Plant Interactions

**Figure 3:**
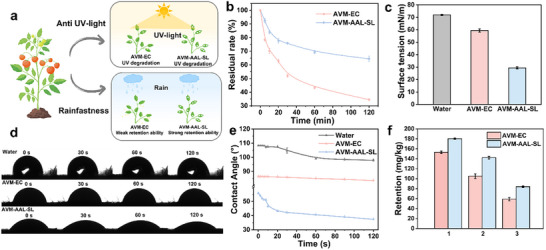
Schematic simulation of photolytic resistance and surface activity a); Photostability b), surface tension c), contact angle images d) and data e), retention after rainfall f) of AVM‐EC and AVM‐AAL‐SL.

The performance of coacervate droplets on plant surfaces, particularly their photostability and wettability, is critical for effective pesticide delivery (**Figure** **3**a). To evaluate these attributes, we conducted simulated experiments assessing the photodegradation resistance and surface activity of the coacervate formulation. Under UV irradiation, the photodegradation profiles of AVM‐EC and AVM‐AAL‐SL followed similar trends, indicating that the coacervate matrix did not alter the intrinsic degradation pathway of abamectin (Figure 3b). However, after 120 min of exposure, AVM‐AAL‐SL retained 64.47% of its active ingredient, compared to just 34.53% for AVM‐EC, demonstrating superior UV stability. The superior UV resistance is achieved through a synergistic mechanism wherein the coacervate architecture physically confines the pesticide in close proximity to the lignin matrix, which in turn actively absorbs and dissipates UV energy, stemming from π‐conjugated aromatic rings that absorb UV radiation, as well as phenolic hydroxyl groups that scavenge reactive oxygen species. The primary mechanism is the intrinsic UV‐absorbing and antioxidant capability of lignin's aromatic constituents, while the coacervate structure serves as an essential enabling framework that maximizes the efficacy of this chemical protection.

Surface tension measurements further highlighted the interfacial advantages of the coacervate system. As shown in Figure 3c, AVM‐AAL‐SL exhibited a markedly lower surface tension (29.40 mN m^−1^) than both AVM‐EC (59.47 mN m^−1^) and water (71.96 mN m^−1^). This reduction is attributed to the amphiphilic nature of the lignin derivatives: anionic SL provides inherent surface activity, while octylamine‐modified AAL increases hydrophobicity. Their electrostatic pairing forms a pseudo‐surfactant complex that promotes tight molecular alignment at the air–liquid interface, thereby significantly lowering surface tension.^[^ ^35^ ^]^ As a result, AVM‐AAL‐SL demonstrated improved spreading on leaf surfaces, as reflected in the contact angle measurements on tomato leaves (Figure 3d,e). Within 120 s, the contact angle of AVM‐AAL‐SL decreased from 56.02° to 37.40°, substantially lower than that of AVM‐EC (86.62° to 83.63°) and water (108.28° to 97.93°), indicating superior wetting and adhesion characteristics.

To mimic real‐world field conditions, repeated rain‐wash simulations were conducted to assess formulation retention (Figure 3f). Even after three rainfall cycles, AVM‐AAL‐SL maintained significantly higher retention than AVM‐EC. This enhanced rainfastness is likely due to hydrogen bonding between phenolic hydroxyls and secondary amines within the coacervate matrix and hydroxyl or carboxyl groups present on the leaf cuticle. Such interactions may anchor the droplets to the leaf surface in a “claw‐like” configuration,^[^ ^36^ ^]^ thereby mitigating the loss of active ingredients during rainfall.

### 2.6 Dynamics and Removal of Pesticide Residues

**Figure 4:**
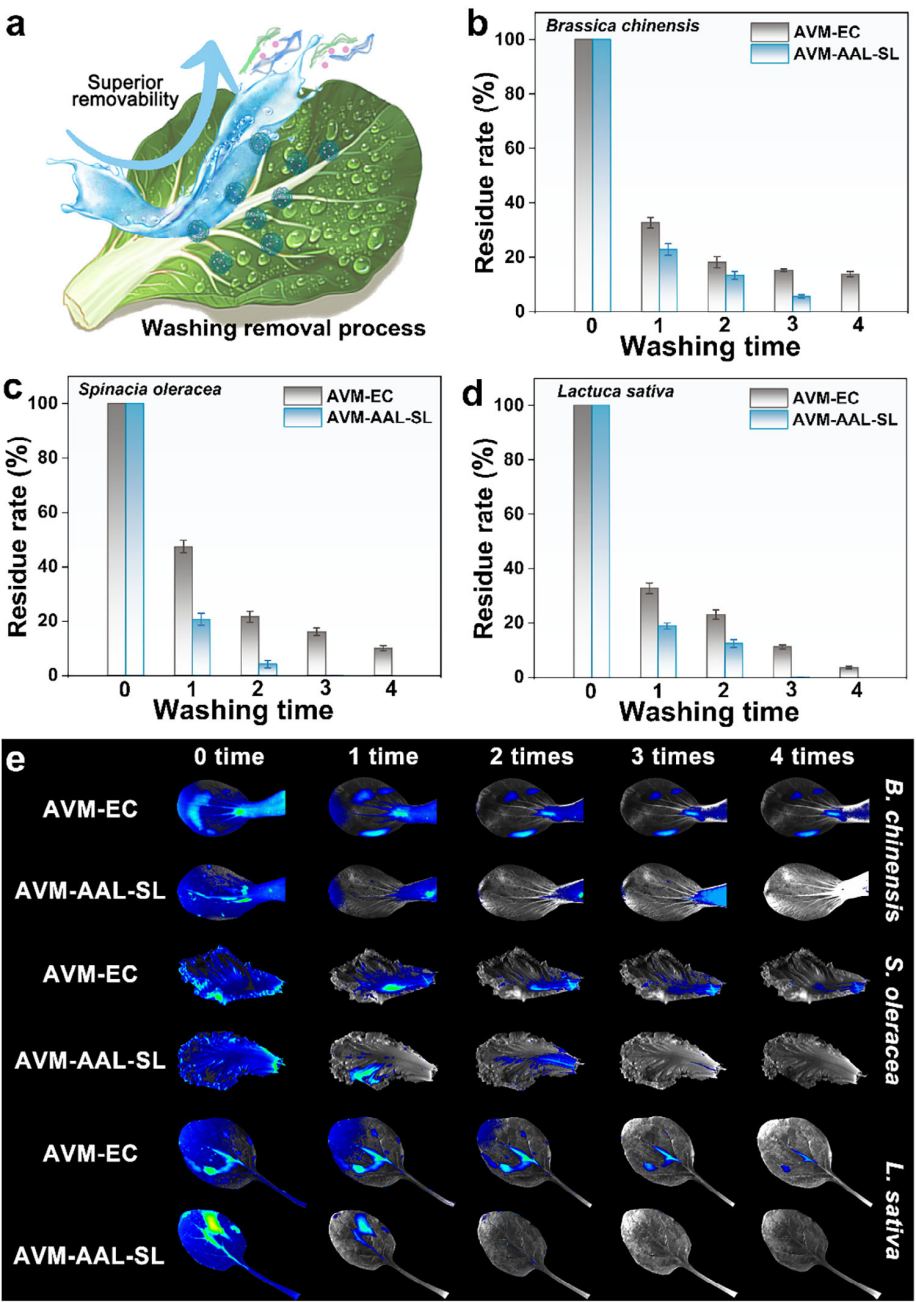
Schematic diagram for washing to remove coacervates a); Residue rates of different formulations on *B. chinensis* b), *S. oleracea* c), and *L. sativa* d) leaves after different number of washing with sodium bicarbonate solution. Fluorescence imaging of leaves bearing formulation residues after different number of washing with sodium bicarbonate solution e).

The development of pesticide formulations often emphasizes improved spreading and adhesion on plant surfaces to enhance efficacy and persistence. However, these same properties can also lead to increased pesticide residues on edible crops, raising food safety and public health concerns.^[^ ^37^ ^]^ Consequently, the ability to rapidly and efficiently remove pesticide residues from vegetable leaves has become an important criterion for evaluating formulation safety.^[^ ^10^ ^]^ In many Asian households, especially in China, simple, additive‐free washing agents such as sodium bicarbonate solution, rice‐wash water, and flour suspension are widely used to clean fruits and vegetables, offering practical and accessible means for residue removal. To assess their effectiveness, this study systematically examined the removal dynamics of two AVM formulations (AVM‐AAL‐SL and the commercial formulation AVM‐EC) on vegetable leaves using these three common cleaning agents (**Figure** **4**a).

High‐performance liquid chromatography (HPLC) analyses provided quantitative confirmation of these observations (Figure 4b–d). A single rinse with any of the three washing agents significantly reduced residue levels, and AVM‐AAL‐SL consistently showed lower residual content compared to AVM‐EC. After four washes with sodium bicarbonate solution, AVM‐AAL‐SL levels fell below the detection limit, while AVM‐EC residues remained above 10% on *B. chinensis* and *S. oleracea*. Rice‐wash water demonstrated even higher efficacy, completely removing AVM‐AAL‐SL within three washes, though trace amounts of AVM‐EC persisted after four (Figure S8a–c, Supporting Information). In contrast, flour suspension proved least effective: even after four rinses, AVM‐AAL‐SL residues were still detectable, and AVM‐EC levels exceeded 10% across all tested leaves (Figure S9a–c, Supporting Information).

Fluorescence imaging of leaf surfaces treated with sodium bicarbonate solution (Figure 4e) showed that fluorescence intensity correlated with residual formulation levels. Prior to washing, all three leaf types exhibited strong fluorescence, indicating substantial surface adherence. With increasing wash cycles, both signal intensity and coverage area decreased markedly, with AVM‐AAL‐SL showing a more rapid reduction than AVM‐EC. After four rinses, AVM‐AAL‐SL residues were nearly undetectable on all leaves, while significant AVM‐EC fluorescence remained, indicating lower removal efficiency.

The contrasting behaviors of the two formulations stem from their distinct physicochemical properties. While AVM‐AAL‐SL adheres to the leaf surface primarily via hydrogen bonding and other reversible intermolecular forces, its lignin‐based matrix remains susceptible to alkaline dissolution and charge repulsion.^[^ ^38^ ^]^ In comparison, AVM‐EC permeates more deeply into the leaf cuticle due to solvent‐mediated infiltration, leading to stronger and more persistent binding.^[^ ^39^ ^]^ Sodium bicarbonate, being strongly alkaline, promotes lignin dissolution and electrostatic disruption, facilitating the disintegration of the AVM‐AAL‐SL coacervate structure. However, its limited solvent action renders it less effective against deeply embedded AVM‐EC residues.

Rice‐wash water and flour suspension rely partially on physical adsorption and displacement mechanisms, both of which are influenced by pH. The mildly alkaline pH of rice‐wash water promotes surface charge disruption and partial lignin dissolution, enabling synergistic removal effects through both chemical and physical pathways. In contrast, the near‐neutral or slightly acidic pH of flour suspension limits lignin degradation and impairs displacement, resulting in poor cleaning efficacy.

From a regulatory perspective, the facile removability of AVM‐AAL‐SL residues has significant practical implications for food safety compliance. Maximum residue limits (MRLs) are established to ensure that dietary exposure to pesticides remains within toxicologically acceptable levels. The ability of a formulation to be effectively removed by common household washing practices directly reduces the potential for consumer exposure, thereby increasing the likelihood that the final commodity will comply with MRLs. This attribute is particularly valuable for leafy vegetables and other produce that are often consumed fresh. Collectively, these findings demonstrate that AVM‐AAL‐SL achieves a desirable balance between strong surface adhesion during application and easy removal during post‐harvest washing. This dual functionality enhances both agricultural performance and consumer safety, providing a practical advantage over conventional formulations in everyday use scenarios.

### 2.7 Smart Response Release

**Figure 5:**
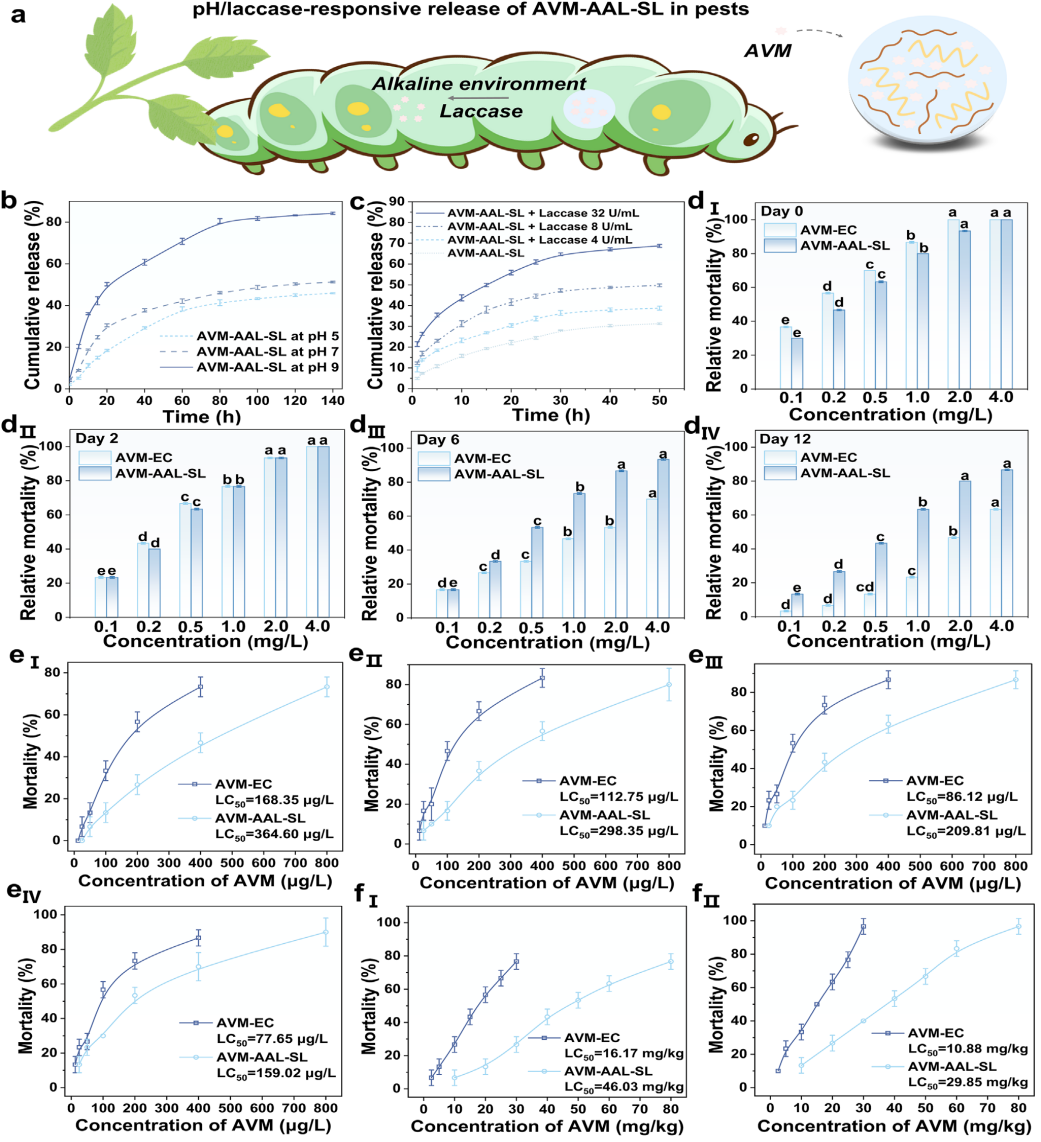
Schematic diagram of pH/laccase‐responsive release of AVM‐AAL‐SL in pests a); Effects of pH b) and laccase (c, at neutral environment) on the release performance of AVM from AVM‐AAL‐SL; Mortality of *Plutella xylostella* after 0 d (d_I_), 2 d (d_II_), 6 d (d_III_) and 12 d (d_IV_) of AVM and AVM‐AAL‐SL treatments; Concentration‐dependent effects of AVM and AVM‐AAL‐SL on the mortality to zebrafish after 24 h (e_I_), 48 h (e_II_), 72 h (e_III_), and 96 h (e_IV_); Concentration‐dependent effects of AVM and AVM‐AAL‐SL on the mortality to earthworm after 7 d (f_I_), and 14 d (f_II_). Different letters indicate statistical differences at *p < 0.05*.

Lignin possesses inherent responsiveness to both pH and enzymatic stimuli, particularly laccase.^[^ ^38^ ^]^ Accordingly, lignin‐based pesticide formulations are expected to exhibit environmentally triggered release behaviors. To evaluate this, the release kinetics of AVM from the AAL‐SL coacervate system were examined under varying pH conditions and laccase concentrations. As shown in **Figure** **5**b, AVM‐AAL‐SL displayed marked pH sensitivity: under alkaline conditions (pH 9), the cumulative release of AVM reached 84.27%, significantly exceeding that observed at neutral (51.27%) and acidic (45.90%) environment. Similarly, the release rate increased with laccase concentration (Figure 5c), reaching 68.7% at 32 U mL^−1^, indicating a clear enzyme‐responsive behavior.

These results can be attributed to the chemical features of the formulation's lignin components. Both aminated alkaline lignin (AAL) and sulfonated lignin (SL) contain pH‐sensitive functional groups, namely phenolic hydroxyls and sulfonic acids, that destabilize under alkaline conditions. The phenolic hydroxyl groups of lignin readily ionize, enhancing its solubility and thus disrupting the original aqueous phase aggregate system. This facilitates the rapid release of encapsulated pesticide.^[^ ^40^ ^]^ In parallel, laccase catalyzes the oxidative degradation of lignin backbones, further accelerating AVM release. This dual‐responsive behavior enables the formulation to remain stable in the external environment while releasing its active ingredient efficiently under specific internal conditions.

Notably, these pH‐ and enzyme‐triggered mechanisms align with the physiological environment of *Lepidopteran* pests, particularly the alkaline midgut and the presence of endogenous laccase‐like enzymes. As illustrated in Figure 5a, this compatibility ensures targeted AVM release within the pest body, thereby enhancing insecticidal efficacy while minimizing off‐target effects.

### 2.8 Biological Activity

Insecticidal activity serves as the primary criterion for evaluating the performance of pesticide formulations in practical applications. The insecticidal efficacy of different AVM formulations was assessed after being sprayed on cabbage leaves for various durations, with results presented in Figure 5d and Table S2 (Supporting Information). As the concentration of the active ingredient in AVM increased, the mortality rate of *Plutella xylostella* also correspondingly rose, with all differences being statistically significant. In contrast, the coacervate droplet formulation, AAL‐SL, exhibited negligible toxicity to *Plutella xylostella*. On Day 0, immediately after treatment, the insecticidal activity of the pure AVM (*LC_50_* = 0.1964 mg L^−1^) was significantly higher than that of the AVM‐AAL‐SL treatment (*LC_50_* = 0.2539 mg L^−1^), which could be attributed to the controlled‐release nature of the coacervated droplets, preventing the rapid release of sufficient AVM active ingredients in the short term. After two days of treatment, the efficacy of AVM showed a noticeable decline (*LC_50_* = 0.2728 mg L^−1^), becoming comparable to that of AVM‐AAL‐SL (*LC_50_* = 0.2815 mg L^−1^). By the sixth day post‐application, AVM‐AAL‐SL still maintained relatively high insecticidal activity (*LC_50_* = 0.4076 mg/L), whereas the activity of AVM, likely due to degradation after application, had significantly diminished (*LC_50_* = 1.2671 mg L^−1^). This decline became even more pronounced by Day 12, with the *LC_50_* of AVM increasing to 2.6177 mg L^−1^, while the *LC_50_* of AVM‐AAL‐SL remained much lower at 0.5903 mg L^−1^. This suggests that the coacervate droplet formulation protects AVM, preventing the degradation of active ingredients, while also imparting sustained‐release characteristics to the formulation, thereby ensuring continuous control of the pests.

### 2.9 Non‐Target Biological Safety

In addition to exhibiting excellent target activity, the impact of a pesticide formulation on non‐target organisms in the environment is also a critical indicator of its efficacy. In this study, zebrafish were selected as the biological model for assessing the acute toxicity of the pesticide to aquatic organisms. As shown in Figure 5e, the toxicity of both AVM and AVM‐AAL‐SL to zebrafish follows a dose‐dependent relationship. The *LC_50_* values for zebrafish in different treatments gradually decreased over time, with the *LC_50_* value for AVM‐AAL‐SL consistently higher than that for AVM. After 96 h of treatment, the *LC_50_* value for AVM was 77.65 µg L^−1^, whereas that for AVM‐AAL‐SL was 159.02 µg L^−1^, indicating that the acute toxicity of AVM‐AAL‐SL to zebrafish was approximately two times lower than that of AVM. Figure 5f showed the toxicity of AVM and AVM‐AAL‐SL to earthworms in soil after 7 and 14 d of treatment. After 7 d, the *LC_50_* value for AVM was 16.17 mg kg^−1^, while that for AVM‐AAL‐SL was 46.03 mg kg^−1^. After 14 d, the *LC_50_* value for AVM decreased to 10.88 mg kg^−1^, while that for AVM‐AAL‐SL was 29.85 mg kg^−1^, a reduction in toxicity by approximately three times. The experimental results prove that the toxicity of AAL‐SL to zebrafish and earthworms can be ignored (Tables S3 and S4, Supporting Information). These results suggested that the coacervate significantly reduces the toxicity of AVM to both zebrafish and earthworms, likely due to the controlled release properties and biocompatibility of AVM‐AAL‐SL, which prevents direct contact between AVM and non‐target organisms, thereby enhancing the safety of the formulation for non‐target species.

Soil, as the ultimate “sink” for environmental pollutants, readily accumulates pesticides after application, creating the potential to impact soil microbial communities. A Venn diagram was used to illustrate the shared and unique operational taxonomic unit (OTU) counts at the OTU level (Figure S10a, Supporting Information). The results indicate that the three treatment groups shared 3279 OTUs, while the CK, AVM‐EC, and AVM‐AAL‐SL groups exhibited 7892, 7721, and 7863 unique OTUs, respectively. Despite the addition of different pesticide treatments, the abundance in the AVM‐EC group slightly decreased, while the AVM‐AAL‐SL group showed minimal difference compared to the CK group, suggesting that the coacervated droplets helped mitigate the detrimental effects of AVM on soil microorganisms. Figure S10b (Supporting Information) displayed a heatmap of the relative abundance differences for the top 50 soil microbial genera, where red indicates higher abundance and blue indicates lower abundance. The results demonstrate uneven distribution of dominant microbial species across the CK, AVM‐EC, and AVM‐AAL‐SL treatments, indicating that both AVM‐EC and AVM‐AAL‐SL may have selective impacts on soil microbial populations. Figure S10c–f (Supporting Information) showed the relative abundance and occurrence frequency of the top ten phyla or genera under different treatments, revealing that, compared to the control, the AVM‐AAL‐SL treatment did not significantly disrupt the major species and abundance of the soil microbial community. In conclusion, environmentally friendly coacervate droplets can alleviate the direct exposure to pesticides, reducing the harmful effects of pesticides on the soil ecosystem.

## 3 Future Work

While this study establishes a proof‐of‐concept for fully lignin‐based coacervates as sustainable pesticide delivery systems, several avenues warrant further investigation to translate this technology from the laboratory to the field.

- Process Scaling and Environmental Fate Profiling. Future efforts will focus on developing a scalable, solvent‐free manufacturing process, potentially via a one‐pot synthesis and spray‐drying, to facilitate industrial production. In parallel, comprehensive long‐term studies on the environmental fate of the coacervates, transformation products, and chronic effects on soil ecosystems—are essential for a complete ecological risk assessment.
- Expanding Functional Versatility and Applicability. The platform's utility will be explored by encapsulating a broader spectrum of agrochemicals (e.g., herbicides and fungicides) with diverse physicochemical properties. Furthermore, we will investigate the incorporation of additional smart triggers, such as pest‐specific enzymes or redox potentials, to enhance targeting precision and functional versatility.
- Field Evaluation and Broader Applicability. Greenhouse trials are a critical next step to validate the efficacy and rainfastness of these formulations under more realistic conditions. It is equally important to extend this delivery platform beyond abamectin to other hydrophobic and hydrophilic pesticides, herbicides, and fungicides, thereby demonstrating its versatility as a universal agrochemical delivery platform.

## 4 Conclusion

In summary, we developed a fully lignin‐based, organic solvent‐free coacervate system composed of sodium lignosulfonate (SL) and alkylamine‐modified alkaline lignin (AAL), achieving spontaneous droplet formation driven by electrostatic and hydrophobic interactions. The resulting AVM‐loaded coacervates (AVM‐AAL‐SL) significantly improved pesticide utilization efficiency through enhanced photostability, adhesion, rain resistance, and pH/laccase‐responsive release tailored to the insect midgut environment. Remarkably, the system also exhibited excellent removability using common household washing solutions, effectively reducing pesticide residues on edible crops, an advantage over conventional formulations. Entirely derived from lignin and free of organic solvents, this multifunctional delivery platform combines environmental sustainability with practical efficacy, offering a promising strategy for safer and greener agricultural applications.

## 5 Experimental Section

### Materials

Alkaline lignin (AL) was obtained from Aladdin Reagent Co., Ltd. (Shanghai, China). Avermectin (97% purity) was supplied by Lier Chemical Co., Ltd. (China). Sodium lignosulfonate (SL, 96% purity), fluorescein isothiocyanate (FITC), and Nile Red were purchased from Rhawn Co., Ltd. (China). Sodium hydroxide (NaOH) and formaldehyde were sourced from Sigma–Aldrich. Laccase (2 U mg^−1^) was provided by Nanjing Duly Biotechnology Co., Ltd. (Nanjing, China). A commercial avermectin emulsion concentrate (5% active ingredient) was obtained from Jinong Crop Protection Ltd. (Jiangxi, China). All other chemicals were of analytical grade and purchased from Sinopharm Chemical Reagent Co., Ltd. (China); they were used without further purification.

### Synthesis of Aminated Alkaline Lignin

To initiate the reaction, 5 g of alkaline lignin (AL) was dissolved in 10 mL of 0.4 mol L^−1^ sodium hydroxide solution under continuous stirring for ≈10 min to ensure complete dissolution. Subsequently, predetermined amounts of amination reagents including aminomethane (C_1_), ethylenediamine (C_2_), propylamine (C_3_), butylamine (C_4_), and octylamine (C_8_), along with 37 percent aqueous formaldehyde solution, were sequentially added to the lignin solution. The resulting reaction mixture was maintained at a specified temperature of 45 °C for a defined reaction time of 5 h, with continuous stirring to promote the mannich condensation. After completion, the reaction products were purified by dialysis using tubing with a molecular weight cut off of 1000 Da to remove small molecular weight impurities. The dialyzed solution was subsequently freeze dried to obtain the aminated lignin samples. The products modified using C_1_, C_2_, C_3_, C_4_, and C_8_ were designated as AAL_1_, AAL_2_, AAL_3_, AAL_4_, and AAL_8_ respectively. The resulting samples were characterized by Fourier transform infrared spectroscopy using a Bruker Vertex 70 V spectrometer (Germany), proton nuclear magnetic resonance spectroscopy using a Bruker Avance III 400 HD instrument (Switzerland), and elemental analysis using a Thermo Flash Smart analyzer (United States).

### Preparation of AAL‐SL Coacervates

SL and AAL were mixed in defined volumes and thoroughly stirred at 25 °C to prepare solutions with varying mass ratios. The morphological features of the resulting mixtures were examined using an optical microscope (XSP 8C or 8CA, Teelen, China) to determine the critical concentration for coacervate formation. Dried droplets on silicon substrates were further analyzed by scanning electron microscopy (S4800, Hitachi, Japan). The zeta potential of the coacervates was measured using a Zeta‐sizer Nano ZS (Malvern Panalytical Ltd, United Kingdom). UV–vis absorption spectra were recorded using a Genesys 180 UV–vis spectrophotometer (Thermo Scientific, USA).

### O‐PTIR Microspectroscopy

O‐PTIR (Optical Photothermal Infrared) microspectroscopy was performed using a *mIRage* microscope (Photothermal Spectroscopy Corp., Santa Barbara, CA). The system employed a dual‐range quantum cascade laser (QCL) operating as the pump source, covering the spectral ranges of 3000–2700 and 914–1800 cm^−1^ within a single unit. The QCL operated at a pulse rate of 100 kHz and 100% power with a 2.5% duty cycle. A 532 nm laser, set at 28% power, served as the probe beam. Detection of the reflected probe signal was carried out using a standard room‐temperature silicon photodiode detector. Spectral data were acquired in reflection mode using an all‐reflective Cassegrain‐style objective (40× magnification, 0.78 numerical aperture, 8 mm working distance). Spectra were collected at a resolution of 6 cm^−1^ with a single scan per replicate, with each scan requiring ≈1 s. A QCL background power spectrum was recorded daily from a clean Kevley low‐E slide. To reduce atmospheric interference, the system was continuously purged with dry nitrogen gas.

### Isothermal Titration Calorimetry

Thermodynamic interactions between AAL and SL were analyzed using a MicroCal iTC200 calorimeter (Malvern Instruments, UK). The sample cell was loaded with 200 µL of buffer solution or AAL solution, while the titration syringe was filled with the SL solution. The titration protocol involved 18 sequential injections of 2 µL each at a constant temperature of 25 °C. The heat changes associated with each injection were recorded in real‐time, and the resulting heat flow data were integrated using *Origin* software to generate enthalpograms. The binding isotherms were subsequently analyzed to derive the molar enthalpy (ΔH) and other thermodynamic parameters characterizing the interaction.

### Phase Diagram

The critical point for phase separation was determined by analyzing the differential turbidity curve. The phase boundary was established based on the critical mass ratio of AAL to SL corresponding to the onset of turbidity. The occurrence of condensate formation, indicative of phase separation, was visually confirmed using an optical microscope (XSP 8C or 8CA, Teelen, China) and a fluorescence microscope (Olympus IX83, Japan).

### Turbidimetric Titration

Turbidity measurements were conducted at a wavelength of 670 nm using a Brinkman PC920 probe colorimeter (Anton Paar, Austria) with temperature controlled at 25.0 ± 0.5 °C. Deionized water was used as the reference and set to zero turbidity. For titration, AAL solutions were prepared at a concentration of 0.5 g L^−1^, and SL solution was similarly prepared at 0.5 g L^−1^. Equal volumes of AAL and SL solutions were mixed at varying mass ratios to evaluate the effect of composition on turbidity and phase separation. For each treatment, AAL solution was gradually added to SL solution under constant stirring, and turbidity values were recorded after the mixture reached a stable equilibrium state, typically within 2 to 3 min. Each measurement was repeated in triplicate to ensure accuracy, and the average values were used to construct the turbidity profile for determining the critical mass ratio corresponding to phase separation.

### Confocal Laser Scanning Microscopy

Confocal fluorescence imaging of AAL‐SL coacervates encapsulating fluorescein isothiocyanate (FITC) and Nile red was performed using an FV1000 IX81 confocal laser scanning microscope (Olympus, Japan). A 20 µL of the coacervate solution was placed on a clean glass slide and covered with a coverslip prior to imaging. Fluorescent dyes were excited at a wavelength of 559 nm. FITC and Nile red emission signals were captured in their respective fluorescence channels to visualize the distribution and encapsulation behavior within the coacervate droplets. All images were acquired under consistent laser power and detector settings to ensure comparability across samples.

### Encapsulation Efficiency for AVM

Encapsulation of AVM was conducted in 2 mL of AAL‐SL coacervate dispersions, with AVM added at final concentrations of 0.005, 0.01, 0.02, 0.04, 0.06, 0.08, and 0.1 mm. To promote encapsulation, the mixtures were subjected to vigorous stirring, ensuring sufficient interaction between AVM and the coacervate matrix. Following mixing, the dispersions were centrifuged at 5000 revolutions per minute for 5 min to separate the coacervate phase from the unencapsulated AVM. The supernatants were then diluted fourfold with deionized water and filtered through a 0.22 µm membrane to remove residual particulates. The concentration of free AVM remaining in the supernatant was quantified by high performance liquid chromatography (HPLC) using an instrument from Agilent Technologies (United States). The encapsulation efficiency (*EE*) was calculated using the following equation:

(1) $$\mathrm{EE}\;\;\left(\%\right)=\left(1-\frac{C_{S}V_{S}}{C_{T}V_{T}}\right)\;\times 100\%$$

where *C_S_* (mm) and V_S_ (µL) denote the concentration and volume of the supernatant, respectively, while *C_T_* (mm) and *V_T_* (µL) represent the initial total concentration and volume of AVM. A C_18_ reversed‐phase column (250 mm × 4.6 mm i.d.) was employed for separation, using a mobile phase composed of methanol and water (85:15, v/v) at a flow rate of 1.0 mL·min^−1^. Detection was performed at the wavelength of 245 nm.

### Dynamics and Removal of Pesticide Residues

To evaluate the ability to rapidly and effectively remove pesticide residues from the surfaces of leafy vegetables, leaves of *Brassica chinensis*, *Spinacia oleracea*, and *Lactuca sativa* (at least ten leaves of comparable size selected for each variety) were individually immersed in 100 mg L^−1^ solutions of AVM‐AAL‐SL and AVM‐EC for 30 s. After air drying, each leaf was rinsed with 5 mL of sodium bicarbonate solution, rice‑wash water, or flour suspension respectively for varying numbers of cycles to assess the efficacy of residue removal.

### Fluorescence Imaging

Leaves treated with Nile red–stained AVM‐AAL‐SL and AVM‐EC were subjected to different washing frequencies using 0.5% sodium bicarbonate solution. Residual pesticide formulations on the leaf surfaces were visualized using fluorescence imaging (excitation wavelength: 530 nm; emission wavelength: 636 nm) with a multifunctional imaging system (Tanon 5200Multi, China). For each vegetable type, five leaves were randomly selected for analysis in each experimental group.

### Quantification of Residual Pesticides

Leaves from various vegetable species, previously treated with either the AVM‑AAL‑SL or AVM‑EC formulation, were evenly divided into treatment groups and washed a predetermined number of times using 5 mL of sodium bicarbonate solution, rice‑wash water, or flour suspension respectively, which were readily available in the household. Subsequently, 5 g of similarly sized leaf tissue, collected from comparable positions, were transferred to 50 mL centrifuge tubes, thoroughly homogenized, and extracted with 10 mL of methanol by vortexing for 5 min. Following centrifugation at 8000 rpm for 10 min, the supernatant was collected, and the extraction repeated twice with fresh methanol. All supernatants were combined and analyzed by HPLC to determine the AVM concentration. Each washing treatment was replicated five times, and the pesticide residue rate (*RR*) was calculated according to the following formula:

(2) $$\mathrm{RR}=\left(C_{0}-C_{n}\right)/C_{0}$$

where *RR* denotes the pesticide residue removal rate (%), *C_0_* denotes the amount of pesticide residue without washing (mg/g), and *C_n_* denotes the amount of pesticide residue after *n* times of washing(mg g^−1^).

### Controlled‐Release Performance of AVM‐AAL‐SL

The release behavior of AVM from AVM‐AAL‐SL coacervates was assessed using a dynamic dialysis approach.^[^ ^41^ ^]^ Briefly, 1 mL of AVM‐AAL‐SL dispersion or AVM ethanol solution (used as the control) was suspended in 20 mL of water and loaded into a dialysis membrane (molecular weight cut‐off: 1000 Da). The dialysis bag was immersed in 150 mL of ethanol/water solution (80:20, v/v) contained in a sealed glass bottle and placed in a shaking incubator maintained at 25 °C with a constant agitation rate of 200 rpm. At predetermined time intervals, 1 mL of the external medium was withdrawn and analyzed for AVM concentration using high‐performance liquid chromatography (HPLC). An equal volume of fresh ethanol/water solution was replenished immediately to maintain sink conditions. Additionally, the effects of varying pH levels and the presence of laccase on AVM release profiles were investigated to simulate different environmental triggers. Each treatment was performed in triplicate.

### Greenhouse Insecticidal Activity

The insecticidal efficacy of AVM‐AAL‐SL under greenhouse conditions was assessed at the experimental greenhouse base of China Agricultural University. Cabbage plants were uniformly sprayed with suspensions containing either AVM‐AAL‐SL or technical‐grade AVM. Leaves were harvested at predetermined time points: 0 days (3 h post‐application), 2 days, 6 days, and 12 days after treatment. The harvested leaves were then provided as feed to third‐instar larvae of the diamondback moth (*Plutella xylostella*). Deionized water‐treated plants served as the negative control. Following a 48‐h feeding period under controlled environmental conditions (25 °C, 65% relative humidity, and a 16 h light/8 h dark photoperiod), larval mortality was recorded. To ensure statistical reliability, ten healthy *P. xylostella* were selected for each treatment, with three replicates per treatment.

### Pesticide–Plant Interactions

Surface tension measurements were conducted using a BCZ‐800 fully automatic interfacial tensiometer (Zibo Benchuang Instrument Co., Ltd.). The instrument was calibrated with deionized water until a stable reading in the range of 72–74 mN·m^−1^ was achieved. Subsequently, AVM‐EC and AVM‐AAL‐SL formulations were transferred into the sample dish to a depth corresponding to one‐third of the height from the dish bottom. After allowing the liquid surface to stabilize, surface tension values were recorded. Each formulation was measured in triplicate to ensure reproducibility.

Contact angle measurements were carried out on plant leaves using an OCA 20 contact angle analyzer (Dataphysics, Germany) equipped with automated droplet deposition and image analysis software. A 2 µL droplet was used for each measurement, conducted at a constant temperature of 25 ± 1 °C. Each formulation was replicated three times.

Rainfastness of the pesticide formulations on *Solanum lycopersicum L*. leaves were assessed gravimetrically. Briefly, 5 g of *S. lycopersicum L*. leaves were arranged behind a microscope slide mounted on a 65° inclined platform. Formulations were sprayed evenly using a manual sprayer. After drying, the samples were washed once, twice and three times respectively with 5 mL of deionized water. Each treatment replicated three times. The mass of pesticide retained per unit mass of *S. lycopersicum L*. leaves were used to evaluate washout resistance.

### Photostability Experiment

AVM‐EC and AVM‐AAL‐SL formulations, containing equivalent concentrations of the active ingredient, were placed in translucent quartz vials and exposed to ultraviolet irradiation at 254 nm using a UV lamp at room temperature. At predetermined time intervals, aliquots were withdrawn, diluted with methanol, and analyzed by high‐performance liquid chromatography (HPLC). Each measurement was performed in triplicate to ensure accuracy and reproducibility.

### Acute Toxicity to Adult Zebrafish

The acute toxicity of AVM‐AAL‐SL formulations to adult zebrafish (*Danio rerio*) was evaluated using a static exposure test. Zebrafish were obtained from Tongzhou Aquarium (Beijing, China) and acclimated for 7 days in an aerated aquaculture system under standardized conditions (25 ± 1 °C, 12 h light/12 h dark photoperiod, and dissolved oxygen maintained above 70% saturation). Following acclimation, fish of uniform size and health status were randomly assigned to test tanks containing different concentrations of AVM‐AAL‐SL, with ten fish per treatment. Deionized water was used as the negative control, while technical‐grade AVM served as the positive pesticide control. Mortality was recorded daily throughout the exposure period to evaluate the acute toxicological effects of the test formulations.

### Nontarget Biosafety in Soil

Avermectin (AVM) and AVM‐AAL‐SL formulations were each prepared at seven different concentration levels. Sample solutions were thoroughly mixed into 500 g of artificial soil to achieve the target concentrations. Deionized water was added to adjust the soil moisture content to ≈30%, and the mixtures were homogenized. Each treatment group was applied to 10 earthworms, with containers covered using breathable gauze. All treatments were performed in triplicate. Earthworm survival and signs of toxicity were recorded on days 7 and 14. Experimental conditions were maintained at 20 ± 2 °C, relative humidity of 80 ± 10%, and continuous illumination at 400–800 lux.

For microbial analysis, soil samples treated with AVM‐EC and AVM‐AAL‐SL at equivalent active ingredient concentrations were collected. Approximately 3 g of soil from each treatment group were placed into sterile tubes, stored at −80 °C, and transported on dry ice for sequencing analysis. The bacterial 16S rRNA gene (V3–V4 region) was amplified using primers 338F (5′‐ACTCCTACGGGGAGGCAGCA‐3′) and 806R (5′‐GACTACHVGGGTWTCTAAT‐3′). High‐throughput sequencing was conducted on an Illumina NovaSeq6000 platform to evaluate microbial α‐diversity and relative species abundance.

### Statistical Analysis

All statistical analyses were performed using DPS version 19.05. Data fitting and curve analysis were carried out using Microsoft Excel 2016 and Origin 2022. Experimental results were presented as the mean ± standard error of the mean based on replicate measurements.

## Conflict of Interest

The authors declare no conflict of interest.

## Author Contributions

X.L. performed conceptualization, wrote the original draft, and data curation. J.T. performed validation. W.Z. performed supervision and funding acquisition. X.Y. performed supervision and funding acquisition. H.W. performed conceptualization, wrote, reviewed, and edited the draft, supervision, project administration, and funding acquisition.

## Supporting information



Supporting Information

## Data Availability

The data that support the findings of this study are available from the corresponding author upon reasonable request.
