# Supplementary material for: Toward Sustainable Pesticide Application: Bridging Efficacy and Food Safety
Source: Adv Sci (Weinh). 2025 Oct 30;13(4):e16248. doi: 10.1002/advs.202516248 (PMC12822399; doi:10.1002/advs.202516248)
Supplement: Supplementary file 1 — Supporting Information [file ADVS-13-e16248-s001.docx]

**Table of the Contents**

**Figure S1.** Optical microscopy images of AAL_1_ and SL with mass ratios of 1:1, 2:1, 3:1, 4:1, 5:1, 6:1, 7:1, and 8:1.

**Figure S2.** Optical microscopy images of AAL_2_ and SL with mass ratios of 1:1, 2:1, 3:1, 4:1, 5:1, 6:1, 7:1, and 8:1.

**Figure S3.** Optical microscopy images of AAL_3_ and SL with mass ratios of 1:1, 2:1, 3:1, 4:1, 5:1, 6:1, 7:1, and 8:1.

**Figure S4.** Optical microscopy images of AAL_4_ and SL with mass ratios of 1:1, 2:1, 3:1, 4:1, 5:1, 6:1, 7:1, and 8:1.

**Figure S5.** Optical microscopy images of AAL_8_ and SL with mass ratios of 1:1, 2:1, 3:1, 4:1, 5:1, 6:1, 7:1, and 8:1.

**Figure S6.** FT-IR spectra of alkaline lignin (AL) and amine-modified alkaline lignin (AAL) (a); ^1^H NMR spectrum of AL and AAL (b).

**Figure S7.** Turbidity curve of AAL-SL solution as a function of the mass ratios of AAL_8_ to SL.

**Figure S8.** Residue rates of different formulations on *B. chinensis* (b), *S. oleracea* (c), and *L. sativa* (d) leaves after different number of washing with rice‑wash water.

**Figure S9.** Residue rates of different formulations on *B. chinensis* (b), *S. oleracea* (c), and *L. sativa* (d) leaves after different number of washing with flour suspension.

**Figure S10.** Venn diagram of bacterial OTUs (a), heat map analysis of the 50 most abundant genera (b), relative abundance at the bacterial top ten phylum (c) and genus (d), OTUs occurrence frequency of the bacterial top ten phylum (e) and genus (f) after the treatment of soil with AVM-EC and AVM-AAL-SL.

**Table S1.** C, H and N element contents of alkaline lignin and aminated lignin.

**Table S2**. Toxicity of AVM-EC, blank AAL-SL and AVM-AAL-SL against *Plutella xylostella* larvae at 0, 2, 6, and 12 days after spraying.

**Table S3.** Median lethal concentration (*LC_50_*) values of AVM-EC, AAL-SL, and AVM-AAL-SL to zebrafish after treatments with different time.

**Table S4.** Median lethal concentration (*LC_50_*) values of AVM-EC, AAL-SL, and AVM-AAL-SL to earthworm after treatments with different time.

**
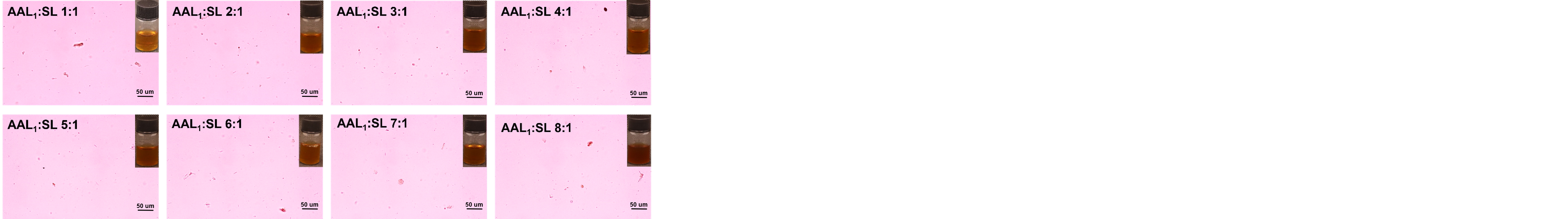
**

**Figure S1.** Optical microscopy images of AAL_1_ and SL with mass ratios of 1:1, 2:1, 3:1, 4:1, 5:1, 6:1, 7:1, and 8:1.

**
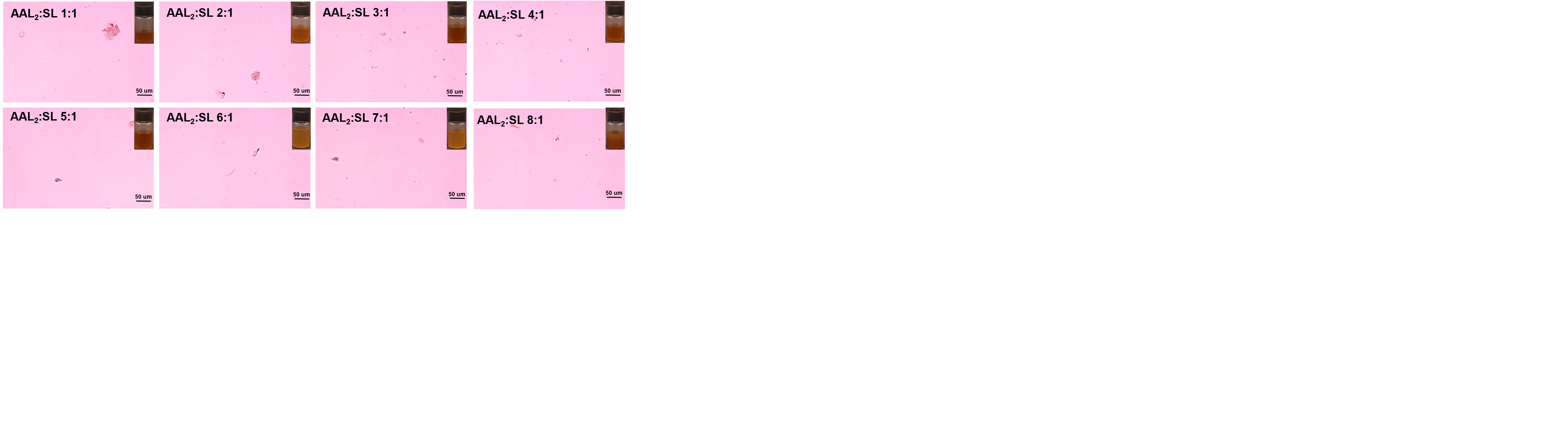
**

**Figure S2.** Optical microscopy images of AAL_2_ and SL with mass ratios of 1:1, 2:1, 3:1, 4:1, 5:1, 6:1, 7:1, and 8:1.


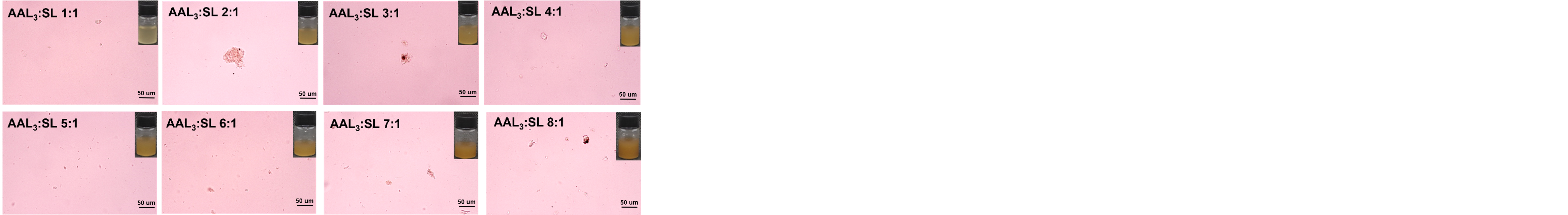


**Figure S3.** Optical microscopy images of AAL_3_ and SL with mass ratios of 1:1, 2:1, 3:1, 4:1, 5:1, 6:1, 7:1, and 8:1.


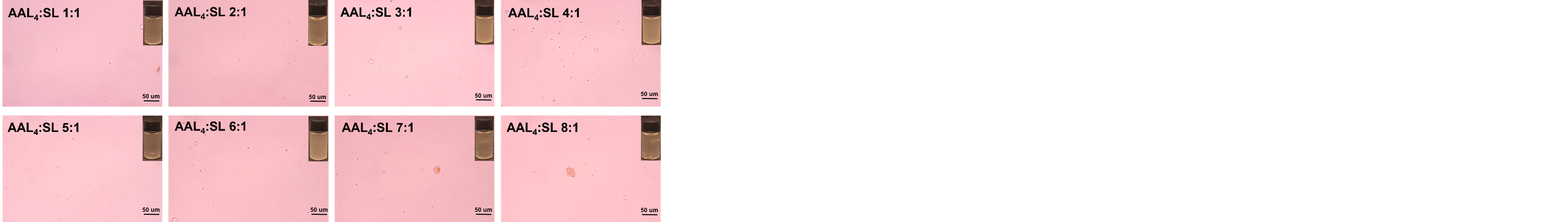


**Figure S4.** Optical microscopy images of AAL_4_ and SL with mass ratios of 1:1, 2:1, 3:1, 4:1, 5:1, 6:1, 7:1, and 8:1.

**
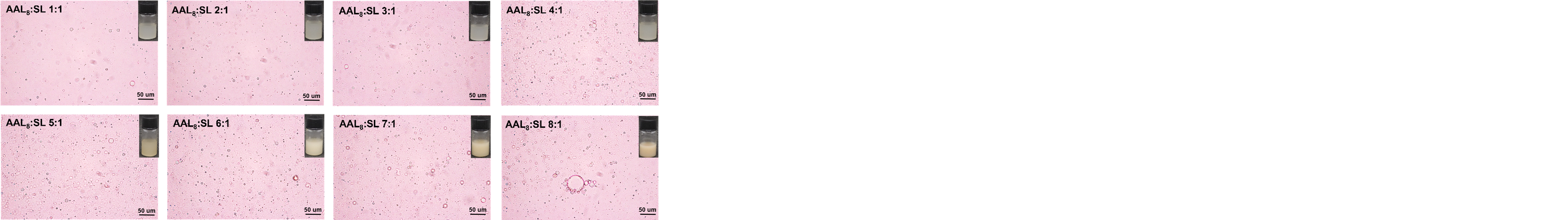
**

**Figure S5.** Optical microscopy images of AAL_8_ and SL with mass ratios of 1:1, 2:1, 3:1, 4:1, 5:1, 6:1, 7:1, and 8:1.

**
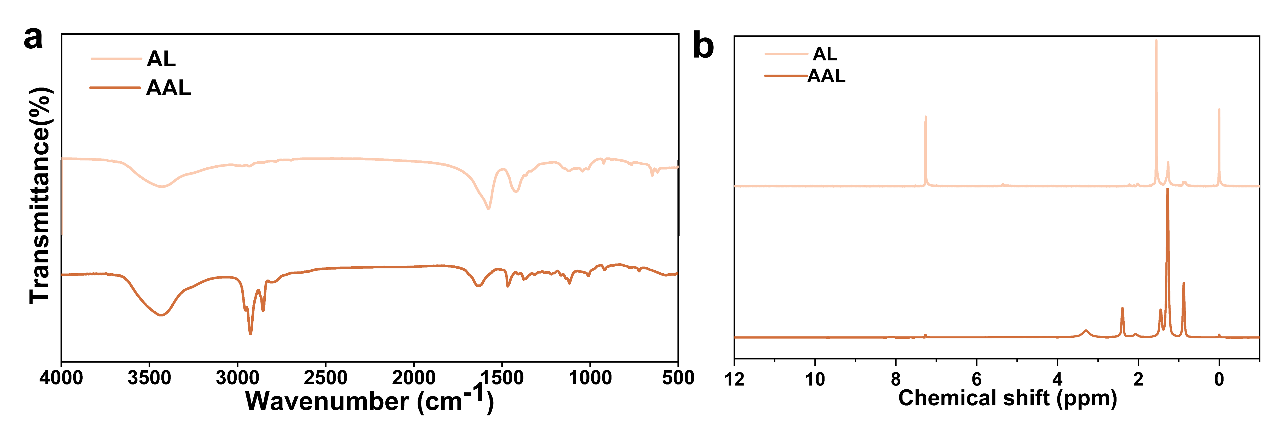
**

**Figure S6.** FT-IR spectra of alkaline lignin (AL) and amine-modified alkaline lignin (AAL) (a); ^1^H NMR spectrum of AL and AAL (b).


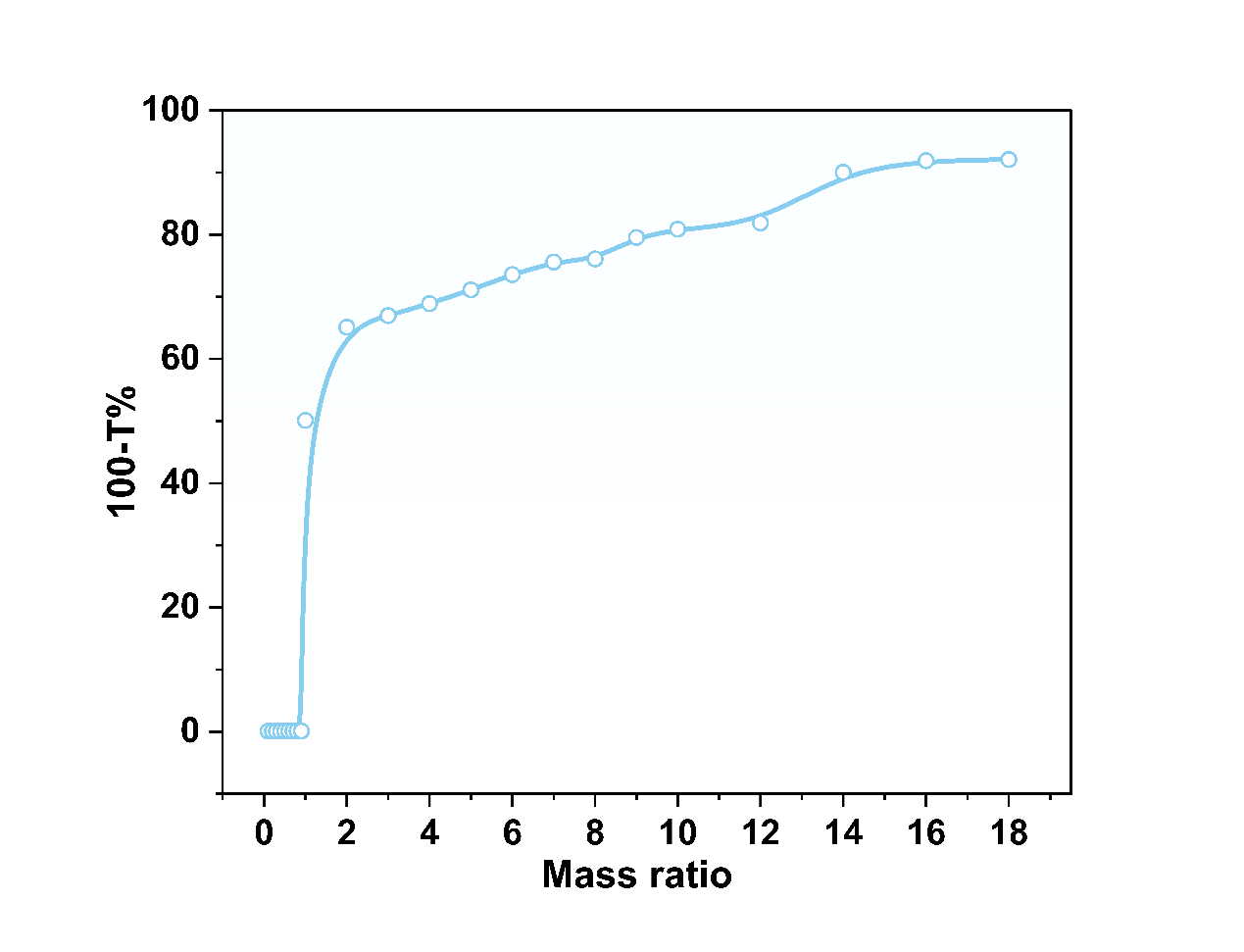


**Figure S7.** Turbidity curve of AAL-SL solution as a function of the mass ratios of AAL_8_ to SL.


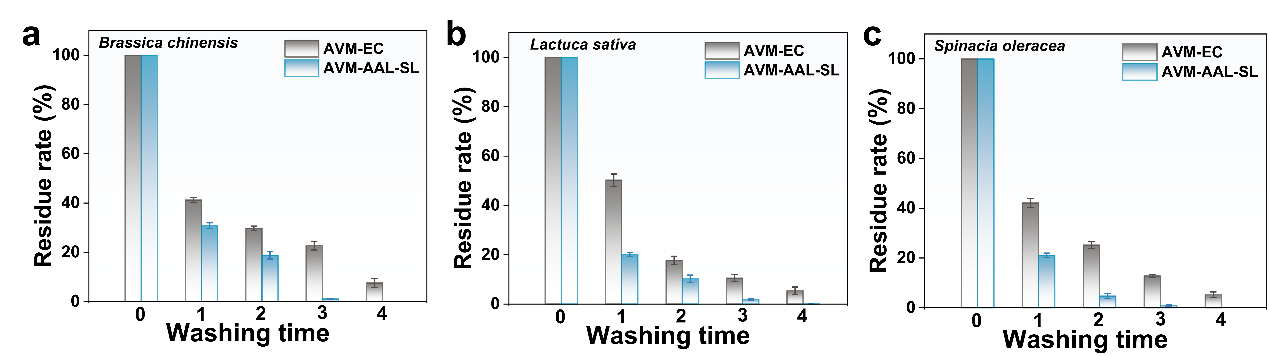


**Figure S8.** Residue rates of different formulations on *B. chinensis* (b), *S. oleracea* (c), and *L. sativa* (d) leaves after different number of washing with rice‑wash water.


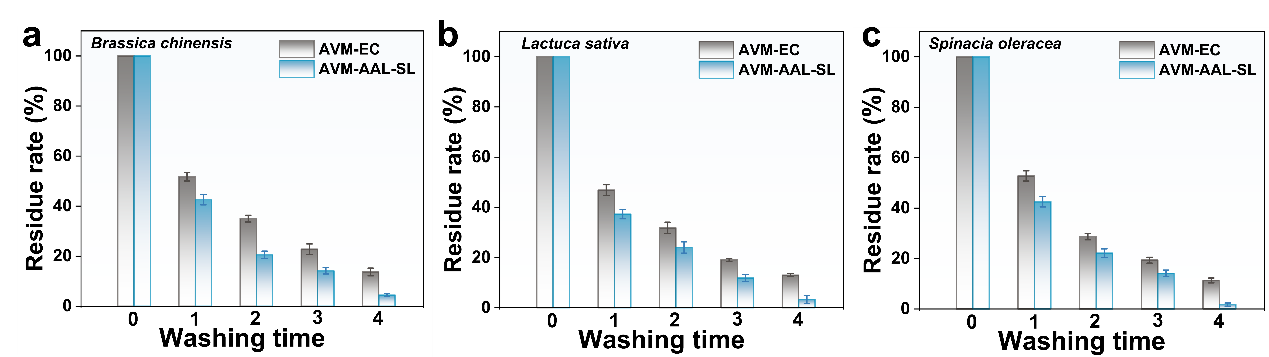


**Figure S9.** Residue rates of different formulations on *B. chinensis* (b), *S. oleracea* (c), and *L. sativa* (d) leaves after different number of washing with flour suspension.


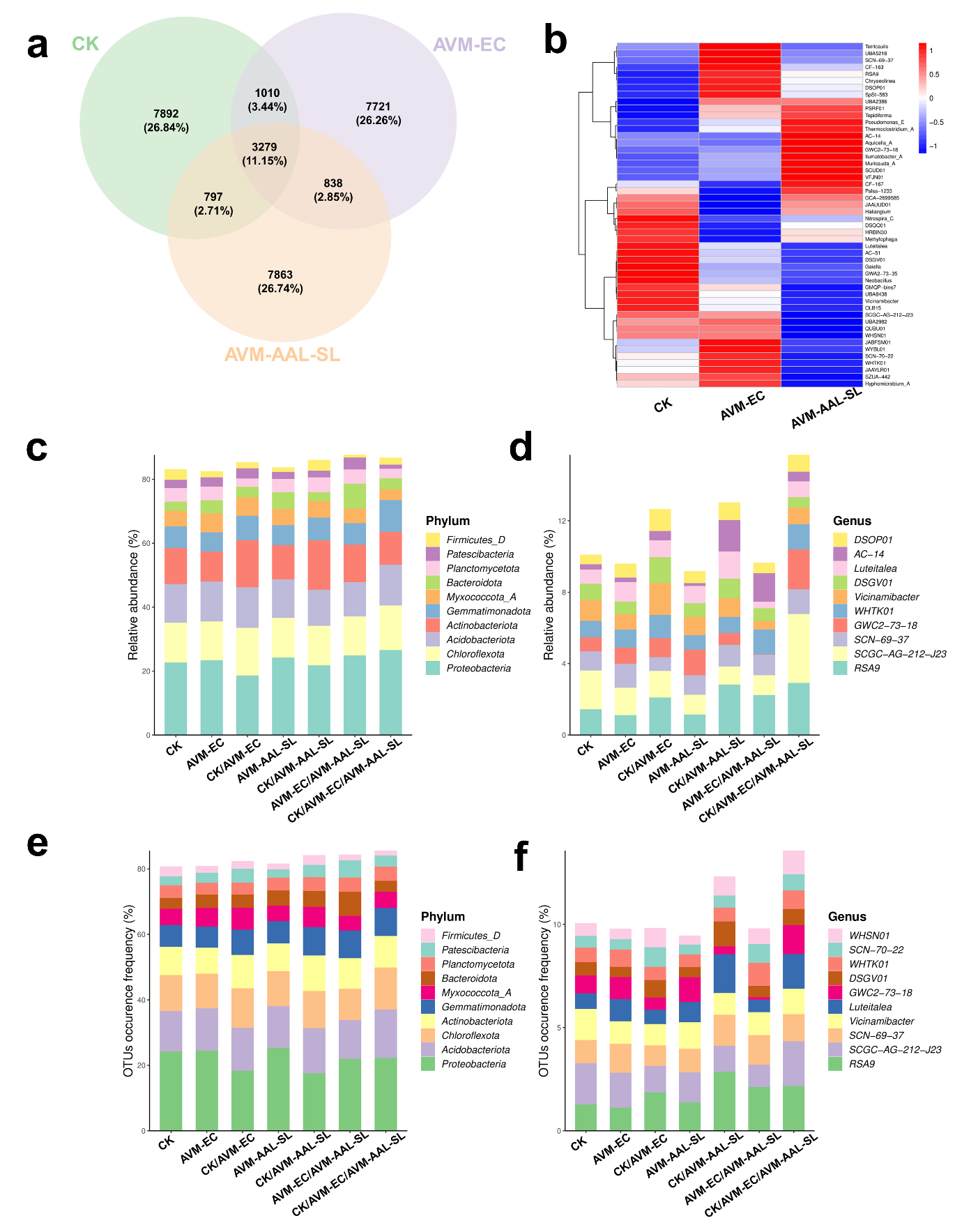
**Figure S10.** Venn diagram of bacterial OTUs (a), heat map analysis of the 50 most abundant genera (b), relative abundance at the bacterial top ten phylum (c) and genus (d), OTUs occurrence frequency of the bacterial top ten phylum (e) and genus (f) after the treatment of soil with AVM-EC and AVM-AAL-SL.

**Table S1.** C, H and N element contents of alkaline lignin and aminated lignin.

| Samples | C (%) | H (%) | N (%) |
| --- | --- | --- | --- |
| Alkaline lignin | 60.41 | 3.99 | 0.40 |
| Aminated lignin | 68.95 | 11.59 | 8.07 |

**Table S2**. Toxicity of AVM-EC, blank AAL-SL and AVM-AAL-SL against *Plutella xylostella* larvae at 0, 2, 6, and 12 days after spraying.

| **Time after**  **spraying (d)** | ***LC_50_* (95% Confidence interval) (mg/L)** | | |
| --- | --- | --- | --- |
|  | **AVM-EC** | **AAL-SL** | **AVM-AAL-SL** |
| **0** | 0.196 (0.0873-0.4419) | 150.47 (135.83-166.68) | 0.254 (0.103-0.627) |
| **2** | 0.273 (0.118-0.631) | 170.43 (156.86-185.17) | 0.282 (0.124-0.641) |
| **6** | 1.267 (1.039-1.545) | 368.36 (337.32-402.25) | 0.408 (0.384-0.433) |
| **12** | 2.618 (2.025-3.383) | 534.63 (417.63-684.42) | 0.590 (0.539-0.647) |

**Table S3.** Median lethal concentration (*LC_50_*) values of AVM-EC, AAL-SL, and AVM-AAL-SL to zebrafish after treatments with different time.

| **Treatment time (h)** | ***LC_50_* (95% Confidence interval) (μg/L)** | | |
| --- | --- | --- | --- |
|  | **AVM-EC** | **AAL-SL** | **AVM-AAL-SL** |
| **24** | 168.35 (82.59-343.17) | 1350.51 (1142.92-1595.81) | 364.604 (171.42-775.52) |
| **48** | 112.75 (94.17-134.99) | 1120.44 (883.98-1420.15) | 298.35 (234.40-379.75) |
| **72** | 86.12 (72.58-102.17) | 962.73 (802.68-1154.69) | 209.81 (162.56-270.78) |
| **96** | 77.65 (62.01-97.24) | 913.65 (782.22-1067.16) | 159.02 (129.55-195.18) |

| **Treatment time (day)** | ***LC_50_* (95% Confidence interval) (mg/kg)** | | |
| --- | --- | --- | --- |
|  | **AVE-EC** | **AAL-SL** | **AVM-AAL-SL** |
| **7** | 16.17 (14.14-18.49) | 891.31 (827.26-960.31) | 46.03 (41.32-51.27) |
| **14** | 10.88 (7.89-15.00) | 800.05 (765.23-836.45) | 29.85 (24.37-36.56) |

**Table S4.** Median lethal concentration (*LC_50_*) values of AVM-EC, AAL-SL, and AVM-AAL-SL to earthworm after treatments with different time.
